# Magnitude and factors associated with surgical site infection among mothers underwent cesarean delivery in Nekemte town public hospitals, western Ethiopia

**DOI:** 10.1371/journal.pone.0250736

**Published:** 2021-04-27

**Authors:** Diriba Ayala, Tadesse Tolossa, Jote Markos, Mekdes Tigistu Yilma

**Affiliations:** 1 Department of Midwifery, Institutes of Health Sciences, Wollega University, Nekemte, Ethiopia; 2 Department of Public Health, Institutes of Health Sciences, Wollega University, Nekemte, Ethiopia; 3 Department of Nursing, Institutes of Health Sciences, Wollega University, Nekemte, Ethiopia; 1. IRCCS Neuromed 2. Doctors with Africa CUAMM, ITALY

## Abstract

**Background:**

Surgical site infection (SSI) is a serious public health problem due to its impacts on maternal morbidity and mortality and it can have a significant effect on quality of life for the patient. However, little has been studied regarding the magnitude and factors associated with SSI among women underwent cesarean delivery (CD) in study area. Therefore, the aim of this study was to assess the magnitude and factors associated with SSI among women underwent cesarean delivery in Nekemte Town Public Hospitals 2020.

**Methods:**

An institution based cross-sectional study was conducted from January 1/2018 to January 1/2020. A simple random sampling technique was employed to select 401 patient cards from all records women underwent CD from January 1/2018 to January 1/2020. Epidata version 3.2 was used for data entry, and STATA version 14 was used for analysis. A logistic regression model was used to determine the association of independent variables with the outcome variable and adjusted odds ratios (AOR) with 95% confidence interval was used to estimate the strength of the association.

**Results:**

Three hundred eight two (382) cards of women were selected for analysis making a response rate of 95.2%. The mean (±SD) age of the mothers was 25.9 (±4.8) years. The prevalence of SSIs was 8.9% (95% CI: 6.03, 11.76). Age > 35 years (AOR = 5.03, 95% CI:1.69, 14.95), pregnancy-induced hypertension (AOR = 5.63, 95%CI:1.88, 16.79), prolonged labor (AOR = 4.12, 95% CI:1.01, 32.19), receiving general anesthesia (AOR = 3.96 95% CI:1.02, 15.29), and post-operative hemoglobin less than 11 g/dl (AOR = 4.51 95% CI:1.84, 11.07) were significantly associated with the occurrence of SSI after cesarean delivery.

**Conclusions and recommendations:**

The magnitude of post CD SSI in this study was comparable with the sphere standards of CDC guidelines for SSI after CD. Concerned bodies should give due attention the proper utilization of partograph to prevent prolonged labor, and provision of iron folate to increase the hemoglobin level of pregnant mothers in all health institution. In addition, we would recommend the use of spinal anesthesia over general anesthesia.

## Introduction

Surgical site infection (SSI) occurs after surgery in the part of the body where the surgery take place and occurred within 30 days after the operation [1, 2]. It is a serious public health problem because its high rates of morbidity and mortality and it can have a significant effect on quality of life, it increase extended hospital stay and financial burden to healthcare providers [3–5].

Despite the magnitude of SSI is lower in high-income countries, it remains the second most frequent type of health care-associated infections in Europe and the USA [6]. According to recent studies conducted in developed countries, the magnitude of SSI was 20% in USA and 17% in Europe [7]. In developing countries, SSI was the most frequently reported healthcare associated infections [8]. According to WHO 2017 annual report, the prevalence of SSI in low and middle income countries (LMIC) was 11.7% [9]. In Sub-Saharan African (SSA) countries, SSI is the most frequent complication in surgery and up to 20% of women who give birth by caesarian delivery developed SSI [4]. The magnitude of SSI in SSA ranges from 2.87% in Egypt [10] to 10.9% in Tanzania [11].

In Ethiopia, SSI after CD still constitutes a major problem to maternal health. A systematic review and meta-analysis findings from Ethiopia showed the SSI after CD was 8.81% [12]. Another retrospective audit and case note review conducted in Ethiopia indicated the magnitude of SSI was 8.6% [13]. Recent report indicated the potential contributing factors for SSI was complex and interrelated. The predictors of SSI after delivery was delayed hospital stay, long duration of the surgical operation, lack of sterilization conditions of the operating room, lack of antibiotic prophylaxis before surgery, premature rupture of membranes, frequently performed vaginal examination, and pre-eclampsia [12, 14–16].

The burden of SSIs related with increased maternal morbidity and mortality, additional costs to health systems, and patients [6]. It also increases the post-operative hospital stay time and also increased treatment time and possible reoperation [17, 18]. In addition, SSI has a significant effect on quality of life for the patient [3]. SSI is a common postoperative complication reported at 3–15% of caesarean deliveries. Post-operative infection is responsible for about 12% of maternal deaths in developing countries [14].

Preoperative bathing, surgical site preparation, surgical hand preparation, adequate use of antibiotics to reduce the risk of infection, and surgical wound irrigation are the main prevention strategies of SSI [5, 19]. Moreover, the Prophylactic antibiotics for all CD was used as general recommendation to prevent SSI and it reduces the risk of infection by up to 75% [20]. A systematic review and meta-analysis study conducted in Ethiopia also recommends the prevention of SSI by minimizing early artificial rupture of membranes and provision of Prophylactic antibiotics [12].

The study on SSI and associated factors are scanty in Ethiopia and not available in study area. In order to improve maternal morbidity and mortality, identifying the magnitude of SSI and associated factors among women underwent CD is paramount importance. This study was aimed to emphasize on the magnitude and associated factors of SSI among women underwent cesarean delivery in Western Ethiopia.

## Methods

### Study area and study design

An Institution-based cross-sectional study was conducted in the Nekemte Public hospitals (Nekemte specialized Hospital and Wollega University Referral Hospital (WURH)). Both hospitals are found in Nekemte town, the capital city of East Wollega Zone. Nekemte is located 331 kilometers from Addis Ababa, the capital city of Ethiopia. Data were retrieved between January 15 to 30, 2020 among women who were delivered by CD from January 1, 2018 –January 1, 2020.

### Population

All women who had delivered by C/S in Nekemte public health facilities were a source population, and study population were all women who were given birth by CD at selected Nekemte town public Hospitals from January 1, 2018–January 1, 2020.

### Eligibility criteria and participants

All cesarean deliveries performed after period of viability were included in the study. Patients who died before the third day of post-operative were excluded, because SSIs cannot be diagnosed before three days of operative procedure. In addition, cards of the client without outcome variable and incomplete data were excluded from the study.

### Sample size determination and sampling technique

The desired sample size was determined for both objectives using single and double population proportion formula and the largest sample size was selected for this study. A single population proportion formula n = (Zα/2)^2^ P (1 − P) ^2^/d^2^ was used to calculate the sample size for the first objective. Assumptions of 8% the population proportion of magnitude of SSI in Debretabor general hospital [21], 95% confidence interval, 5% of marginal error, and 5% non-response rate was used to calculate sample size. Using these data, the desired sample size with the non-response rate was, n = **119.**

For the second objective, double population proportion formula was used in EPI INFO version 3.2 by considering different significant variables from previous study [21]. Sample size was determined using a formula for two population proportions by taking the level of significance to be 5% and the power 80%. Duration of operation (≥30 min) was considered as risk factor of SSIs. P1 = Proportion of women with a duration of operation ≥30 min which was 11.4%. P2 = Proportion of operation with < 30 min which was 3.8%. r is ratio of population among exposed to non-exposed and it was 1:1. Based on the above assumptions, and 5% non- response rate, **401** women who gave birth by CS were calculated as a final sample size.

**Fig 1:**
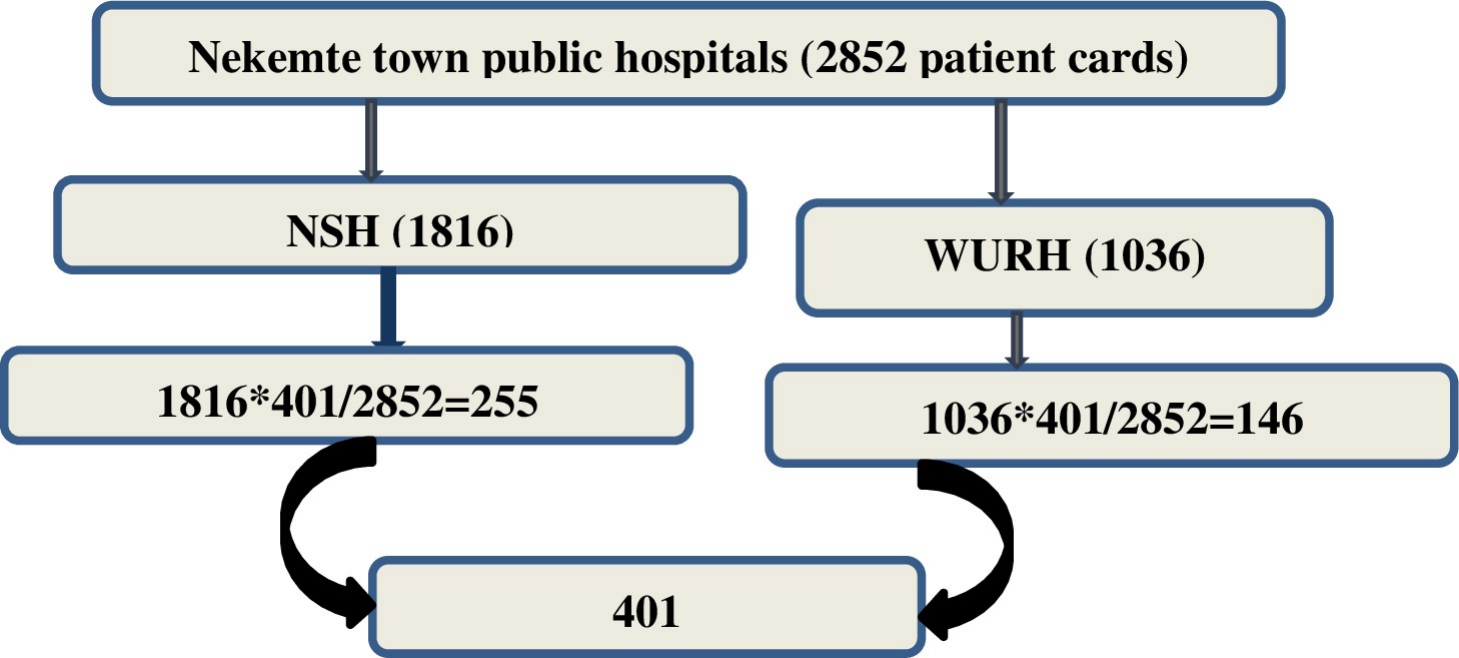
Schematic presentation of sampling procedure.

This study was collected from two public hospitals of Nekemte town (Nekemte specialized hospital and WURH). Then the sample size was proportionally allocated to each hospital. Individual unique card number was retrieved from their operation register, and the number was used to find their medical cards. The card number of all women those gave birth by CD at two hospitals from January 1, 2018 –January 1, 2020 was added to sampling frame. Finally, computer generated simple random sampling technique was employed to select samples from each hospital (Fig 1).

### Outcome measurement

The outcome variable was SSI (Yes/No). SSI is an infection that developed after cesarean section on the operational site/near, which is diagnosed by health professional/ clinician. Surgical site infection status was recorded as “Yes” if the women were diagnosed for surgical site infection after CD and “No” if the women were not diagnosed for surgical site infection after CD.

Independent variables were Socio-demographic factors of the women (age and residence), medical and obstetrics related factors (parity, gestational age, onset of labor, status of membrane, duration of labor, number of vaginal examination, antenatal care follow-up, chorioaminionitis, pregnancy induced hypertension, DM, and chronic hypertension). Furthermore, operation related factors such as indication of CD, prophylaxis, type of anesthesia, type of cesarean section, duration of the operation, skin incision type, skin suturing type, estimated blood loss, intra operative blood transfusion, and post-operative hemoglobin level were also included as independent variables.

### Data collection tools, procedure and quality assurance

Data was collected from the registers of hospitals using checklists developed specifically for the study from operation logbook and medical cards. The checklist consists of socio-demographic data, obstetrics and medical condition of the women, and operation related variables. Data were collected from medical record of women. The outcome variable SSI was confirmed as “Yes/No” by reviewing medical or operation registration book at the hospitals, which was recorded by health professionals. Two trained midwifes working in operation and delivery room was conducted data extraction and the principal investigator of the research was supervised the overall data collection activity. A pre-test was conducted on 5% of patient records at WURH before the actual data collection to assess the clarity of the checklist. Based on the findings of pretest, possible amendments were made (variable such as Per-operative hemoglobin level was removed from the checklist).

### Data management and analysis

Epi data version 3.2 was used for data entry, and then the data was exported to STATA version 14 for further analysis. Descriptive statistics such as frequency, percentage, mean and standard deviation were computed. Before analysis, data were cleaned and edited by using simple frequencies and cross-tabulation. Before fitting the variable in the model, the assumption of the logistic regression model was checked. The binary logistic regression model was fitted to determine factors associated with SSI. Factors that were associated with the outcome variable at P-value <0.25 significant level in the bivariable logistic regression analysis were included in the multivariable logistic regression analysis. Then crude odds ratio (COR) and adjusted odds ratio (AOR) together with their corresponding 95% confidence intervals was presented in the final multivariable logistic regression table. Statistical significance was declared at a 5% level (p-value < 0.05).

### Ethical approval

Ethical approval was obtained from the ethical review committee of Wollega University, Institute of Health and support letter was written to each hospital. In addition, Ethical Clearance Committee waived the requirement for informed consent to have data from the patient medical records. Then, Nekemte specialized hospital and Wollega University referral hospital wrote a permission letter to respective delivery ward and operation room. As the study was conducted through a review of medical records, patient confidentiality was kept. Moreover, no personal identifier was used on the data collection checklist.

## Results

### Socio demographic and medical characteristics of the women

Three hundred eighty-two (382) cards of women were selected for analysis making a response rate of 95.2%. Nineteen (19) cards were excluded from analysis because their cards were not available. The mean (±SD) age of the mothers was 25.9 (±4.8) years. The minimum and maximum age was 18 and 40 years old. Majority 199 (51.1%) of mothers were found in the age group of 25–34 years, while 35 (9.2%) of participants were aged ≥35 years old. The study revealed that 212 (55.5%) of women resides in rural area Among 382 women who underwent cesarean section, 4 (1.0%) of the women were HIV positive and three (0.5%) women who had preexisting diabetes mellitus. Only 0.8% of the women had chronic hypertension (Table 1).

**Table 1:** Socio-demographic and medical characteristics of the women who underwent c/s in Nekemte town public hospitals, Ethiopia from 2018 to 2020 (N = 382).

Variables	Category	SSI		Chi2	P value	Total No (%)
		Yes No (%)	No No (%)			
**Age**	≤24	9 (26.5)	139 (39.9)	37.90	0.00	148 (38.7)
	25–34	12 (35.3)	187 (53.7)			199 (51.1)
	≥35	13 (38.2)	22 (6.4)			35 (9.2)
**Residence**	Urban	13 (38.2)	157 (45.1)	0.59	0.44	170(44.5)
	Rural	21 (61.8)	191 (54.9)			212 (55.5)
**HIV Status**	Reactive	0(0.0)	4 (1.1)	0.92	0.53	4 (1.0)
	Non-reactive	34 (100.0)	344 (98.9)			378 (99.0)
**Chronic HTN**	Yes	0 (0.0)	3 (0.9)	2.22	0.14	3 (0.8)
	No	34 (100.0)	345 (99.1)			379 (99.2)
**DM**	Yes	0 (0.0)	2 (0.6)	0.19	0.67	2 (0.5)
	No	34 (100.0)	341 (99.4)			380 (99.5)

**No**- Number, **HTN**- Hypertension, **DM**- Diabetes Mellitus

### Obstetrics characteristics of women

Two hundred ten (55%) of the women were multipara, and 20 (5.2%) were grand multipara. Almost all women (95.3%) had antenatal care (ANC) follow-up, only two mothers had gestational diabetes mellitus. Forty-five (11.8%) mothers were developed pregnancy induced hypertension. Majority of women 357 (93.5%) underwent caesarian delivery while they were at term. Two hundred ninety-seven (77.7%) operation was performed while the women were on labor, and around 130 (34%) of the women stayed on labor more than 24 hours before operation (Table 2).

**Table 2:** Obstetrics characteristics of the women who underwent CD in Nekemte town public hospitals, western Ethiopia from 2018 to 2020 (N = 382).

Variables	Category	SSI		Chi2	P value	Total No (%)
		Yes No (%)	No No (%)			
**Parity**	Premiparous	11(32.4)	141 (40.5)	11.67	0.00	152 (39.8)
	Multipara	17 (50.0)	193 (55.5)			210 (55.0)
	Grand multipara	6 (17.6)	14(4.0)			20 (5.2)
**ANC**	Yes	28 (82.4)	336 (96.5)	0.29	0.59	364 (95.3)
	No	6 (17.6)	12 (3.5)			18 (4.7)
**Gestational age at C/S**	Preterm	7 (25.6)	18 (5.2)	0.32	0.56	25 (6.5)
	Term	27 (74.4)	330 (94.8)			357 (93.5)
**Gestational DM**	Yes	0 (0)	2 (0.6)	0.2	0.66	2 (0.5)
	No	34 (100)	346 (99.4)			380 (99.5)
**Pregnancy induced hypertension (PIH)**	Yes	12 (35.3)	33 (9.4)	19.86	0.00	45 (11.8)
	No	22 (64.7)	315 (90.6)			337 (89.2)
**Mother has been in labor before operation**	No labor	6 (17.6)	79 (22.7)	2.82	0.24	85 (22.3)
	≤24hrs	12 (35.3)	155 (44.5)			167 (43.7)
	>24hrs	16 (47.1)	114 (32.8)			130 (34.0)
**Vaginal Examination done**	No exam	3 (8.8)	25 (7.2)	3.28	0.19	28 (7.3)
	1–3 times	11 (32.4)	169 (48.6)			180 (47.1)
	≥4 times	20 (58.8)	154 (44.2)			174 (45.6)
**Membrane state**	Intact	8 (23.5)	170 (48.9)	7.98	0.00	178 (46.6)
	Ruptured	26 (76.5)	178 (51.1)			204 (53.4)
**Duration of membrane rupture**	≤24hrs	10 (38.5)	79 (43.9)	5.84	0.02	89 (43.2)
	>24hrs	16 (61.5)	101 (56.1)			117 (56.8)
**Chorioaminonitis**	Yes	1 (2.9)	13 (3.7)	0.01	0.92	14 (3.7)
	No	33 (97.1)	335 (97.3)			368 (96.3)

**No**- number, **ANC**- Antenatal care, **DM**- Diabetes mellitus, **C/S**- cesarean section

### Operation related characteristics of the women

All women who underwent cesarean delivery had received antibiotics prophylaxis. About 54 (14.1%) and 328 (85.9%) of the procedures were elective and emergency surgery, respectively. Of 382 participants, 357 (93.5%) were received spinal anesthesia. Regarding the type of abdominal incision performed, 349 (91.4%) was pfannenstiel incision. About 122, (31.2%) of the participants had less than 500 ml intra-operative blood loss, and only 7.6% of the skin closure was interrupted (Table 3).

**Table 3:** Operation related characteristics of the women who underwent CD in Nekemte town public hospitals, western Ethiopia from 2018 to 2020 (N = 382).

Variables	Category	SSI		Chi2	P-value	Total No (%)
		Yes No (%)	No No (%)			
**Type of C/D**	Emergency	28 (82.4)	300 (86.2)	0.38	0.54	328 (85.9)
	Elective	6 (17.6)	48 (13.8)			54 (14.1)
**Type of anesthesia used**	General	6 (17.6)	19 (5.5)	7.52	0.00	25 (6.5)
	Spinal	28 (82.4)	329 (94.5)			357 (93.5)
**Type of skin incision**	Pfannensteil	29 (85.3)	320 (92.0)	1.74	0.19	349 (91.4)
	Midline	5 (14.7)	28 (8.0)			33 (8.6)
**Duration of Procedure**	≤60 min	25 (73.5)	310 (89.1)	6.94	0.00	335 (87.7)
	>60 min	9 (26.5)	38 (10.9)			47 (12.3)
**Estimation of blood loss**	<500 ml	7 (20.6)	115 (33.0)	2.21	0.14	122 (31.9)
	≥500 ml	27 (79.4)	233 (67.0)			260 (68.1)
**Blood transfused**	Yes	9 (26.5)	45 (12.9)	4.68	0.03	54 (14.1)
	No	25 (73.5)	303 (87.1)			328 (85.9)
**Type of skin closure**	Interrupted	5 (14.7)	24 (6.9)	2.69	0.10	29 (7.6)
	Sub-cuticular	29 (85.3)	324 (93.1)			353 (92.4)
**Post-operative hgb**	<11g/dl	18 (52.9)	51 (14.6)	30.68	0.00	69 (18.1)
	≥11g/dl	16 (47.1)	297 (85.4)			313 (81.9)

**S/D**- Cesarean delivery, **SSI**- Surgical Site Infection, **No**-Number

### Magnitude of surgical site infection

Of the total 382 study participants who underwent cesarean section, 34 (8.9%) of them had developed SSI. Non-reassuring fetal heart rate pattern 173 (45.3%), previous scar of C/S 68 (17.8%), and cephalo-pelvic disproportion 57 (14.9%) were the common indication for cesarean section.

### Risk factors of surgical site infection

#### Bivariable and multivariable logistic regression analysis

During binary logistic regression analysis, those variables that had P-value **<** 0.25 significance level were considered in multiple binary logistic regression. Out of 21 independent variables, 10 variables were selected for multivariable analysis at p-value < 0.25. In multivariable logistic regression, age of the women, prolonged labor, pregnancy-induced hypertension, post-operative hemoglobin level, and type of anesthesia have significant predictors for SSI at p-value ≤ 0.05.

Accordingly, the odds of developing SSI were 5.03 times higher among mothers who had aged ≥ 35 years than those mothers who were age less than 24 years (AOR = 5.03, 95% CI:1.69, 14.95). Mothers with pregnancy-induced hypertension were 5.63 times more likely to develop SSI than those mothers without pregnancy-induced hypertension (AOR = 5.63, 95% CI: 1.88, 16.79). The odds of developing SSI were 4.12 times higher among mothers who were stayed on labor for more than 24 hours before CD than their counter parts (AOR = 4.12, 95% CI: 1.01, 32.19). The odds of developing SSI were 3.96 times higher for those mothers who had received general anesthesia as compared to those mothers who undergone operation with spinal anesthesia (AOR = 3.96, 95% CI: 1.02, 15.29).

Finally, the odds of developing SSI were 4.51 times higher for mothers who had less than 11g/dl hemoglobin than women who had greater 11g/dl hemoglobin level (AOR = 4.51, 95% CI: 1.84, 11.07) (Table 4).

**Table 4:** Multivariable logistic regression analysis for risk factors associated with SSI in Nekemte town public hospitals, western Ethiopia from 2018 to 2020 (N = 382).

Variables	Category	SSI		COR	AOR	P-value
		Yes No (%)	No No (%)			
**Age**	≤24	9 (26.5)	139 (39.9)	Ref	Ref	
	25–34	12 (35.3)	187 (53.7)	0.99 (0.40–2.41)	0.89 (0.33–2.44)	0.835
	≥35	13 (38.2)	22 (6.4)	9.12 (3.48–23.87)	**5.03 (1.69–14.95)**	0.004*
**PIH**	Yes	12 (35.3)	33 (9.4)	5.20 (2.36–11.46)	**5.63 (1.88–16.79)**	0.002*
	No	22 (64.7)	315 (90.6)	Ref	Ref	
**labor before operation**	No labor	6 (17.6)	79 (22.7)	Ref	Ref	
	≤24hours	12 (35.3)	155 (44.5)	0.97 (0.36–2.81)	2.15 (0.49–9.34)	0.307
	>24hours	16 (47.1)	114 (32.8)	0.22 (0.69–4.929)	**4.12 (1.01–32.19)**	0.048*
**Post Hgb**	<11 g/dl	18 (52.9)	51 (14.6)	6.55 (3.13–13.67)	**4.51 (1.84–11.07)**	0.001*
	≥11 g/dl	16 (47.1)	297 (85.4)	Ref	Ref	
**Vaginal exam**	No exam	3 (8.8)	25 (7.2)	Ref	Ref	
	1–3 times	11 (32.4)	169 (48.6)	0.54 (0.14–2.07)	0.55 (0.08–3.68)	0.542
	≥4 times	20 (58.8)	154 (44.2)	1.08 (0.29–3.91)	0.53 (0.05–4.82)	0.580
**anesthesia**	General	6 (17.6)	19 (5.5)	3.71 (1.37–10.04)	**3.96 (1.02–15.29)**	0.040^*^
	Spinal	28 (82.4)	329 (94.5)	Ref	Ref	
**skin incision**	Pfannensteil	29 (85.3)	320 (92.0)	Ref	Ref	
	Midline	5 (14.7)	28 (8.0)	1.97 (0.70–5.49)	1.27 (0.26–6.01)	0.760
**Duration of procedure**	≤60 min	25 (73.5)	310 (89.1)	Ref	Ref	
	>60 min	9 (26.5)	38 (10.9)	2.93 (1.27–6.75)	2.53 (0.91–7.01)	0.073
**skin closure**	Interrupted	5 (14.7)	24 (6.9)	0.42 (0.15–1.21)	0.81 (0.14–4.51)	0.813
	Sub-cuticular	29 (85.3)	324 (93.1)	Ref	Ref	
**EBL**	<500 ml	7 (20.6)	115 (33.0)	Ref	Ref	
	≥500 ml	27 (79.4)	233 (67.0)	1.90 (0.80–4.50)	1.39 (0.50–3.88)	0.64

**AOR**: Adjusted Odds Ratio; **COR**: Crude Odds Ratio; *statistically significant at p<0.05, **EBL**: Estimated blood loss

## Discussion

Determining the magnitude of SSI and identifying its determinants have paramount importance in decreasing the maternal morbidity and mortality. This is very important in a low-income setting such as Ethiopia where maternal mortality and morbidity it high, low infrastructure and fragile health system of country. This study was conducted to determine the magnitude of SSI and its risk factors among women underwent CD in western Ethiopia. A total of 382 selected records of CD women who gave birth by CS from January1, 2018—January1, 2020 were included in the final analysis.

This study revealed that the prevalence of surgical site infection after CD was 8.9% (95% CI: 6.03–11.76) among mothers who delivered by CD. The finding is comparable with previous studies conducted in Ethiopia and other developing countries. Accordingly, it is comparable with study conducted in Assela teaching referral hospital (9.4%) [18], Jimma University Specialized Hospital (11.4%) [11], Debretabor General Hospital (8%) [22], Zewditu Memorial Hospital (8.4%) [21], Hawassa University Teaching and Referral Hospital (11.0%) [21], Felegehiwot referral hospital (7.8%) [21], Ayder Comprehensive and Specialized Hospital (11.7%) [9], and Lemlem Karl hospital (6.8%) [23]. The finding is also similar with other studies conducted in different countries such as Eritrea (6.8%) [23], Tanzania (10.9%) [11], Nigeria (9.1%) [24], Rwanda (10.9%) [25], Kosovo (9.85%) [26], Norway (8.9%) [20], and Sierra Leone (10.9%) [27].

The prevalence of SSIs observed in the current study was slightly less than studies conducted in Malaysia (18.8%) [1] and Uganda (15.5%) [28]. This could be explained by difference in the type the data used for analysis. Most of the previous studies were performed directly by collecting data from the patients, and the current study used a secondary data that may underestimate the prevalence of SSI due to unrecorded outcome.

The prevalence of current study was higher than the result reported from different studies, which indicated the magnitude of SSI 2.8% in Egypt 2.87% [10], 2.1% in Kuwait [29], and 4.25% in Gujarat, 4.5% in in China [30], 2.4% in Peru [31], and 1.44% in Brazilian [32]. The possible reasons for the discrepancy could be due to difference in socio-economic status and health policy.

The study also identified different risk factors associated with SSI following CD such as age of the mother, pregnancy induced hypertension, prolonged labor, type of anesthesia and post-operative hemoglobin. In this study, age had statistically significant association with SSI in which women with age greater than or equal to 35 years had at higher risk of developing SSI. This finding was in line with previous report finding in a selected government Hospitals in Addis Ababa, Ethiopia [33], in Wolaita Sodo University Teaching and Referral Hospital [34], in Indian [35], and in Kosovo [26]. This might be due to the age is one of non-modifiable risk factor that influence wound healing process and reduce the ability of cell growth and repair, which is inevitable as age increases. Furthermore, gradual changes in endocrine and immune system with aging might increases the likelihood of a positive surgical outcome. In contrast to this finding, study conducted in Assela teaching referral hospital reported the increased risk of SSIs in young women [18]. The discrepancy may be due over representation of young age population.

The odds of SSI were 5.6 times higher among women who had PIH than their counter parts. This finding is consistent with the findings of previous studies done in Debretabor General Hospital, Ethiopia [22], Indian [35], and Tanzania [11]. The possible explanation might be hypo-perfusion of the wound caused by peripheral vasoconstriction effect of PIH. In addition, those mothers with such problems might have edematous wound edges responsible for further entry of organisms and establishment of infection.

This study also found that prolonged labor prior to surgery had increased the odds of SSI following CD. This was also consistent with different studies conducted in Ethiopia hospitals [16, 18, 36–38], and a systematic review and meta-analysis in Ethiopia also support the findings [39]. This finding is also similar with other studies conducted in different countries such as Eritrea [23], Egypt [10], Uganda [28] and Nigeria [24]. This relationship between duration of labor and SSI may be due to increased exposure time for infection and the fact that as duration of labor increase, number of vaginal examinations also increased, and this increases the likelihood of acquiring infections.

The other factor that showed significant association was type of anesthesia used for CD. Those mothers whom operations were under general anesthesia were almost 4 times to have SSIs compared to mothers under spinal anesthesia. This finding was in agreement with the study conducted in Ethiopia [12] and a systematic review and meta-analysis done in China [30].

The odds of SSI were 4.5 times higher among women whose post-operative hemoglobin level was less than 11 g/dl than women with greater than or equal to 11 g/dl. This finding is consistent with the findings of studies done in different setting in Ethiopia [21, 37, 38]. This could be due to hypo-perfusion of the wound secondary to anemia and reduced post-operative ambulation. In general, low hemoglobin concentration reduces the oxygen tension in the wound and increases the risk of wound infection by compromising the activity of macrophages and delaying wound healing progress.

### Limitations of the study

First, this study is retrospective review of records. The data obtained from the record was limited due to unregistered and limited information regarding basic socio- demographic and clinical characteristics, which may affect the result of the study. Second, the true prevalence of SSI may be underestimated or overestimated because of some cases with CD might be died before three days or developed SSI after they had discharged from hospital. The study also shares the limitations of cross-sectional studies, which decreases the causal relationship between independent factors and outcome variables.

### Conclusions and recommendations

The magnitude of post CD SSI in this study was comparable with the sphere standards of CDC guidelines for SSI after CD. The study older age group, pregnancy induced hypertension, prolonged labor, type of anesthesia used for CD and post-operative hemoglobin level had showed significant association with post CD SSI. Thus, we would recommend all concerned bodies to stress on counseling of clients on advantages of iron folic acid supplementation to increase hemoglobin level of mother, because post-operative hemoglobin level was another factor contributing for SSI development. Furthermore, to decrease the rate of SSI, health institutions should have to monitor for proper utilization of partograph to prevent prolonged labor, provision of iron folate to increase the hemoglobin level of pregnant mothers in all health institutions. Lastly, socio-demographic and pregnancy related factors were commonly associated with SSI, thus, we would recommend further prospective study should be conducted to clearly identify the relationship between these factors and to determine other factors, which were not studied in this research.

## Supporting information

S1 Dataset (DTA)

## Abbreviations

AOR: Adjusted Odds Ratio
CI: Confidence Interval
COR: Crude Odds Ratio
CD: cesarean delivery
SSI: surgical site infection
DM: Diabetes mellitus
OR: Odds Ratio
PIH: Pregnancy Induced Hypertension
WHO: World Health Organization
